# A Comprehensive and Systematic Analysis Revealed the Role of ADAR1 in Pan-Cancer Prognosis and Immune Implications

**DOI:** 10.1155/2023/7620181

**Published:** 2023-02-21

**Authors:** Jianlin Zhu, Jianjian Zheng, Jinjun Zhang, Songyu Wang, Lu Wang, Yan Zhao

**Affiliations:** ^1^Department of Dermatologic Surgery, The First Affiliated Hospital of Fujian Medical University, Fuzhou, Fujian, China; ^2^Department of Urology, Xianyou County General Hospital, Putian, Fujian, China; ^3^The First Department of Surgery, Xianyou County General Hospital, Putian, Fujian, China; ^4^Institute of Precision Cancer Medicine and Pathology and Department of Pathology, School of Medicine, Jinan University, Guangzhou, Guangdong, China

## Abstract

Adenosine deaminase RNA specific 1 (ADAR1) has been identified as an enzyme that deaminates adenosine within the dsRNA region to produce inosine, whose amplification reinforced the exhaustion of the immune system. Although there were currently cellular and animal assays supporting the relationship between ADAR1 and specific cancers, there was no correlation analysis that has been performed at the pan-cancer level. Therefore, we first analyzed the expression of ADAR1 in 33 cancers based on the TCGA (The Cancer Genome Atlas) database. ADAR1 was highly expressed in most cancers, and there was a closely association between ADAR1 expression and prognosis of patients. Furthermore, pathway enrichment analysis revealed that ADAR1 was involved in multiple antigens presenting and processing inflammatory and interferon pathways. Moreover, ADAR1 expression was positively correlated with CD8^+^ T cell infiltration levels in renal papillary cell carcinoma, prostate cancer, and endometrial cancer and negatively correlated with Treg cell infiltration. In addition, we further found that ADAR1 expression was closely associated with various immune checkpoints and chemokines. Meanwhile, we observed that ADAR1 may be involved in the regulation of pan-cancer stemness. In conclusion, we provided a comprehensive understanding of the oncogenic role of ADAR1 in pan-cancer, and ADAR1 might serve as a new potential target for antitumor therapy.

## 1. Introduction

Despite advances in surgery, chemotherapy, and radiotherapy, the global morbidity and mortality of malignant tumors are on the rise [1]. Tumors remodel the tumor immune microenvironment (TIM) through various factors, such as dysfunction of immune checkpoints and secretion of chemokines [2]. Although there are advances in immunotherapy drugs targeting PD-1, PD-L1, and CTLA4, only a minority of patients benefit from immunotherapy [3]. The prognostic model constructed by combining multiple genes has been used to evaluate the efficacy of tumor immunotherapy, but there is no rigorous clinical proof [4]. Therefore, the exploration of appropriate immunotherapeutic targets is particularly urgent.

ADAR1, adenosine deaminase RNA specific 1, is the main RNA-editing enzyme responsible for the deamination of adenosine to produce inosine (A-to-I) [5]. ADAR1 also participates in a variety of biological processes in a non-RNA-editing manner, the most important of which is the creation of protein-protein interactions through the double-stranded RNA-binding domain (dsRBD) of ADAR1, which directly and systematically regulates protein-based immunity response [6]. As an enzyme, ADAR1 edits endogenous double-stranded RNA (dsRNA), making it unstable and unable to be recognized by nucleic acid sensors (NAS), thereby closing interferon-stimulated gene (ISG) expression and downregulating interferon (IFN) expression [7]. Previous research demonstrates that ISG-positive tumor cells are uniquely susceptible to ADAR1 deficiency, which sensitises tumor cells to immunotherapy and overcomes resistance to checkpoint blockade [8]. The depletion of ADAR1 in cancer cells was susceptible to death by inflammation [9]. ADAR1 prevented the pathway to immune and translational catastrophe by blocking dsRNA activation.

In this study, we evaluated the expression and variation of ADAR1 and analyzed the relationship with the prognosis of patients. We investigated the relationship between ADAR1 and immune cell infiltration, immune suppressor genes, and chemokines. Our findings provided a new insight into the functional role of ADAR1 in pan-cancer and highlighted that ADAR1 may serve as a new potential target for cancer immunotherapy.

## 2. Methods

### 2.1. Data Collection and Handling

The TCGA Research Network (https://portal.gdc.cancer.gov/) has analyzed clinical and molecular data from over 10,000 oncology patients in 33 countries, covering 33 different tumor types and over 10,000 oncology patients. Transcriptomic RNA-seq data for 33 cancers were extracted from TCGA database. 33 cancer types were included: adrenocortical carcinoma (ACC), bladder urothelial carcinoma (BLCA), breast invasive carcinoma (BRCA), colon adenocarcinoma (COAD), lymphoid neoplasm diffuse large B cell lymphoma (DLBC), esophageal carcinoma (ESCA), glioblastoma multiforme (GBM), head and neck squamous cell carcinoma (HNSC), kidney chromophobe (KICH), kidney renal clear cell carcinoma (KIRC), kidney renal papillary cell carcinoma (KIRP), acute myeloid leukemia (LAML), brain lower grade glioma (LGG), liver hepatocellular carcinoma (LIHC), lung adenocarcinoma (LUAD), lung squamous cell carcinoma (LUSC), ovarian serous cystadenocarcinoma (OV), pancreatic adenocarcinoma (PAAD), prostate adenocarcinoma (PRAD), pheochromocytoma and paraganglioma (PCPG), rectum adenocarcinoma (READ), skin cutaneous melanoma (SKCM), stomach adenocarcinoma (STAD), testicular germ cell tumors (TGCT), thyroid carcinoma (THCA), thymoma (THYM), uterine corpus endometrial carcinoma (UCEC), cholangiocarcinoma (CHOL), cervical squamous cell carcinoma and endocervical adenocarcinoma (CESC), mesothelioma (MESO), sarcoma (SARC), uveal melanoma (UVM), and uterine carcinosarcoma (UCS).

### 2.2. Genomic Modifications of ADAR1 in Cancer Patients

Alterations in ADAR1 in cancer patients were obtained from the online cBioPortal database (http://www.cbioportal.org/). Cancer genomic alterations in ADAR1 include copy number amplification, profound deletions, missense mutations of unknown significance, and mRNA upregulation [10].

### 2.3. ADAR1 Protein Expression Analysis

The Human Protein Atlas (HPA: https://www.proteinatlas.org/) database was used to explore the protein levels of ADAR1 in human tumors and normal tissues [11]. The STRING (https://string-db.org/) database was used to construct a protein-protein interaction network (PPI) for ADAR1 [12].

### 2.4. Survival Analysis of the Prognostic Value of ADAR1

The Kaplan-Meier (KM) survival analysis was performed to identify different survival outcomes between the two group differences. The univariate Cox regression model was applied to determine the favourable or unfavourable prognosis of ADAR1. The KM analysis was performed by the R packages “survminer” and “survival” and by the R packages “survival” and “forestplot.”

### 2.5. GSEA

GSEA is a method to analyze the function or pathway of target genes affecting tumor genetics. The Kyoto Encyclopedia of Genes and Genomes (KEGG) database and HALLMARK database were adopted in the R package “clusterProfiler” for GSEA [13, 14]. The significant enrichment results were demonstrated on the basis of net enrichment score (NES), gene ratio and *p* value. Gene sets with NES > 1, NOM *p* < 0.05, and FDR *q* < 0.25 were considered to be significantly enriched.

### 2.6. Correlation Analysis between ADAR1 Expression and Immune Function

TISIDB (http://cis.hku.hk/TISIDB/) is a web server that integrates multiple heterogeneous data types for tumor and immune system interactions [15]. We used the “Lymphocyte,” “Immunomodulator,” and “Chemokine” modules in TISIDB to analyze the correlation between ADAR1 expression and the level of immune infiltration, immune checkpoints, and chemokines for multiple cancer types in TCGA database.

### 2.7. Pan-Cancer Stemness Index Analysis

Sangerbox (http://vip.sangerbox.com/home.html) is a bioinformatics online analysis website; we used the tumor stemness and gene expression modules to analyze ADAR1 expression and tumor stemness index online; the results are presented in the form of barplot and column chart.

### 2.8. Statistical Analysis

Gene expression data from TCGA database were analyzed by Student's *t*-test. The expression of ADAR1 was correlated with the abundance scores of immune cells assessed using Spearman's correlation analysis. All analyses were performed with R software (version 4.1.1, http://www.r-project.org) loaded with R packages (“ggplot2,” “ggpubr,” “limma,” “survival,” “survminer,” “clusterProfiler,” “ESTIMATE,” “enrichplot,” and “forestplot”). *p* < 0.05 was considered statistically significant to provide confidence in the data analysis.

## 3. Results

### 3.1. The mRNA Expression Level and Copy Number Variation of ADAR1 in Pan-Cancer

**Figure 1:**
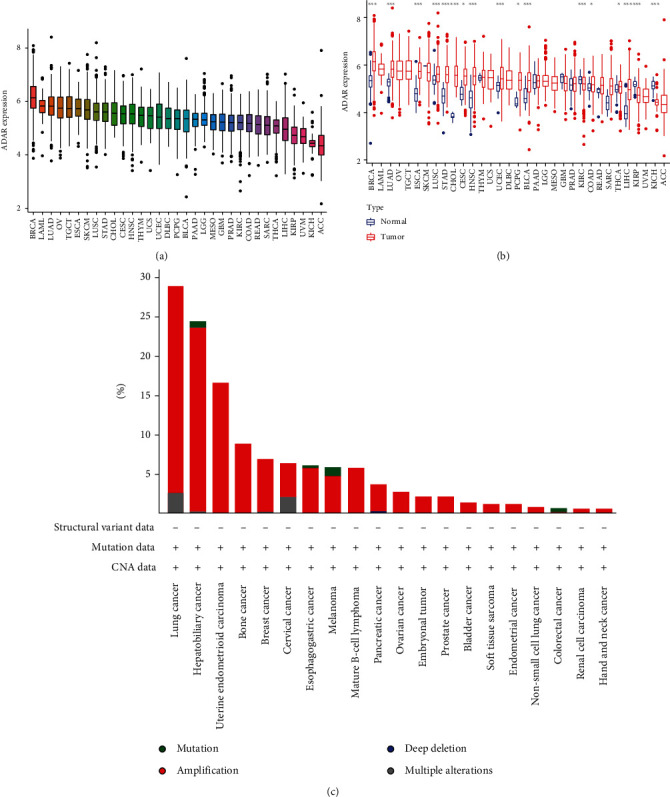
The transcription expression levels and copy number variation (CNV) of ADAR1 in pan-cancer. (a) Ranking of ADAR1 mRNA expression levels in pan-carcinoma tissues. (b) ADAR1 mRNA expression levels in pan-cancer tissues and paired normal tissues. (c) The CNV of ADAR1 in pan-cancer.

We first evaluated the expression of ADAR1 in 33 cancers.  Figure 1(a) shows the ranking of ADAR1 expression in cancer tissues from top to bottom. Subsequently, we observed the expression level of ADAR1 in different cancers. The results showed that ADAR1 was significantly upregulated in 14 cancers, including BRCA, LUAD, ESCA, LUSC, STAD, CHOL, CESC, HNSC, UCEC, PCPG, BLCA, COAD, THCA, and LIHC (Figure 1(b)). It is widely recognized that gene copy number affects the level of gene expression [16]. Therefore, we further analyzed ADAR1 copy number variation (CNV) by the cBioPortal database. We observed that most cancers with CNV exhibited copy number amplification (Figure 1(c)).

### 3.2. Protein Level and PPI Network Analysis of ADAR1 in Pan-Cancer

**Figure 2:**
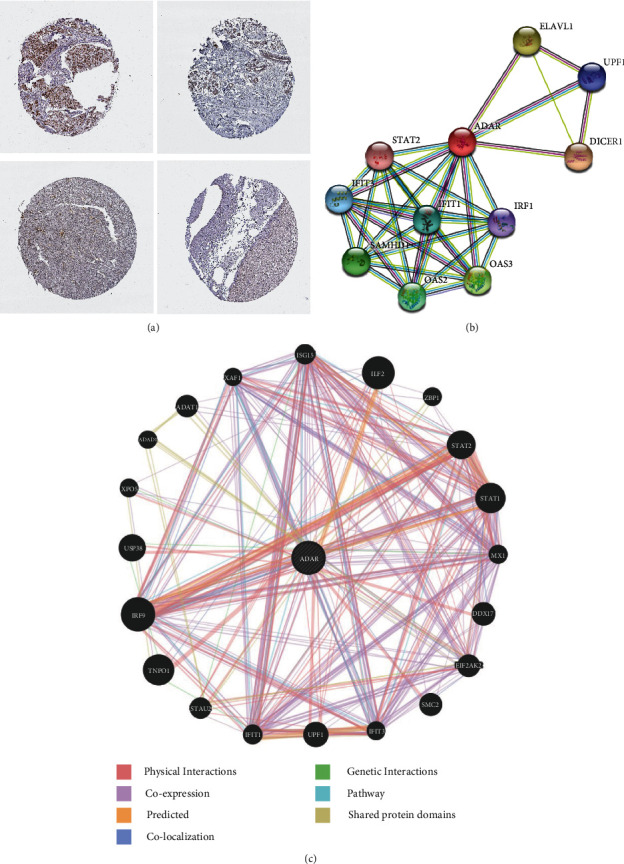
Immunohistochemical and protein-protein interactive modulation network of ADAR1. (a) Immunohistochemistry of ADAR1 in breast and liver cancer and their paired normal tissues. (b) The protein-protein interaction regulatory network of ADAR1 in STRING site. (c) The protein-protein interaction regulatory network of ADAR1 in the GeneMANIA site.

We previously analyzed mRNA expression level and the copy number of ADAR1 in pan-cancer. Next, we further investigated the protein level of ADAR1 in pan-cancer. The result showed that the protein levels of ADAR1 were highest in breast cancer and lung cancer by HPA database. (Figure 2(a) and Supplementary Figure 1). As a potential immunomodulatory gene, a location on the cell membrane is essential. To explore the potential role of ADAR1 in pan-cancer, we constructed a PPI network of ADAR1 via the GeneMANIA and STRING databases. Results from two databases showed that ADAR1 may interact with STAT2, IFIT1, and IFIT3 (Figures 2(b) and 2(c)). Given that STAT2, IFIT1, and IFIT3 play a role in regulating immune cell function, we firmly believed that ADAR1 may be involved in the regulation of the TIM [17, 18].

### 3.3. The Prognostic Significance of ADAR1 in Pan-Cancer

**Figure 3:**
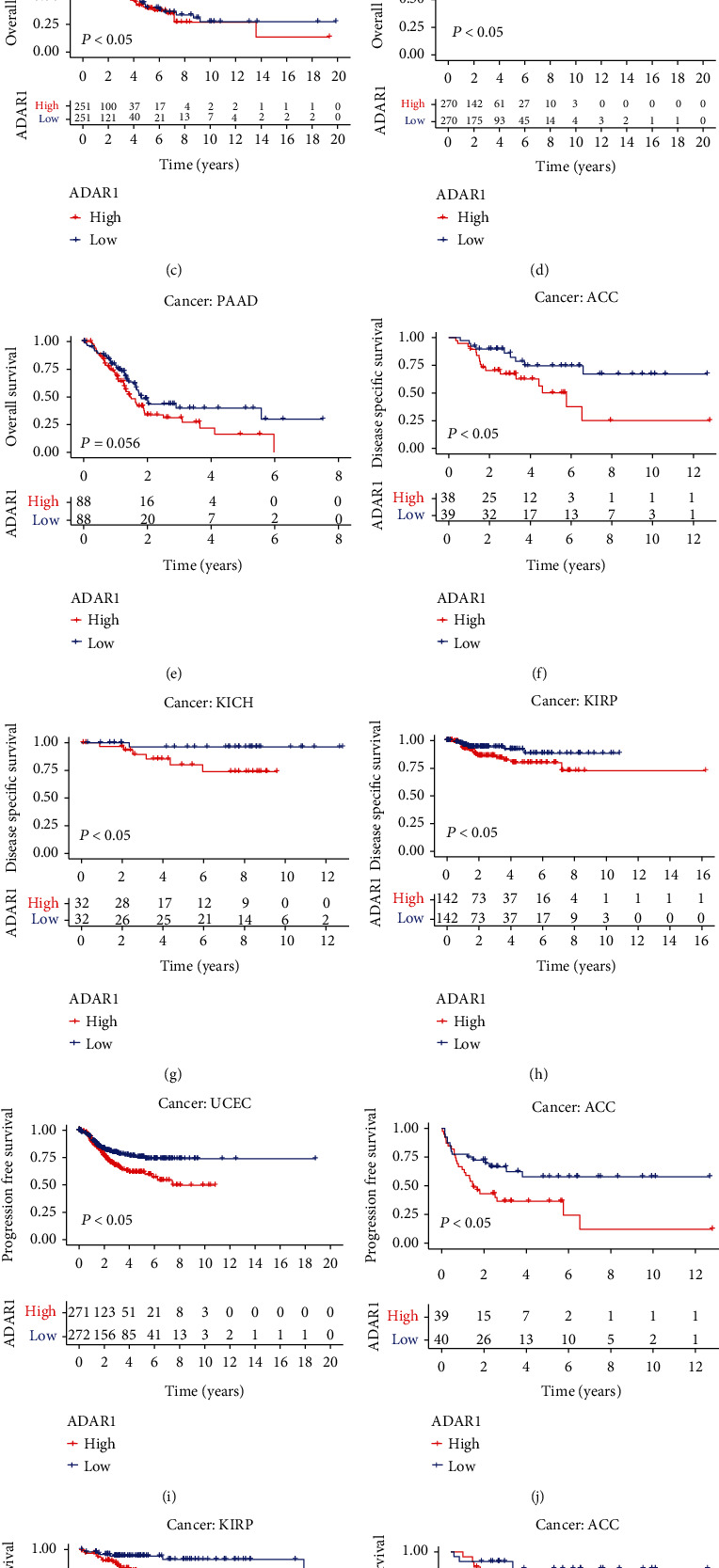
Prognostic analysis of OS and DSS of ADAR1 in different cancers. (a–e) OS analysis of high and low ADAR1 expression in different tumors. (f–h) DSS analysis of high and low ADAR1 expression in different tumors. (i and j) PFS analysis of high and low ADAR1 expression in different tumors. (k and l) DFS analysis of high and low ADAR1 expression in different tumors.

**Figure 4:**
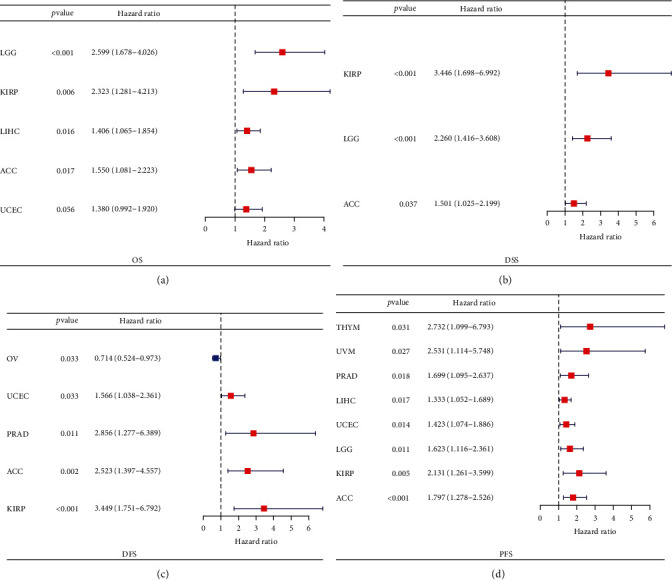
Univariate Cox regression analysis of OS, DSS, DFS, and PFS of ADAR1 in pan cancer. (a) OS, (b) DSS, (c) DFS, and (d) PFS.

We further evaluated the prognostic value of ADAR1 in pan-cancer. We first plotted the KM curves by the median value of ADAR1 expression; the results showed that high expression of ADAR1 in ACC, LGG, LUAD, PAAD, and UCEC had poor overall survival (OS) (Figures 3(a)–3(e)). Interestingly, patients with high ADAR1 expression in ACC, KICH, and KIRP had poor disease-specific survival (DSS) (Figures 3(f)–3(h)). Likewise, UCEC and ACC patients with high ADAR1 expression had poor progression-free survival (PFS), and KIRP and ACC patients with high ADAR1 expression had poor disease-free survival (DFS) (Figures 3(i)–3(l)). In addition, we further analyzed the expression of ADAR1 in the four aspects of OS, DSS, DFS, and PFS by the univariate Cox analysis. The results showed that ADAR1was a risk factor for patients with LGG, KRIP, LIHC, ACC, and UCEC in terms of OS (Figure 4(a)). Meanwhile, DSS analysis revealed that ADAR1 served as a risk factor for patients with KIRP, LGG, and ACC (Figure 4(b)). The DFS analysis revealed that ADAR1 served as a risk factor for patients with UCEC, PRAD, ACC, and KIRP but may be a protective factor for OV (Figure 4(c)). The PFS analysis revealed that ADAR1 served as a risk factor for patients with ACC, KIRP, LGG, UCEC, LIHC, PRAD, UVM, and THYM (Figure 4(d)). These data strongly suggested that ADAR1 plays an important role in tumor prognosis.

### 3.4. Enrichment Analysis of ARDR1 Expression in Pan-Cancer

**Figure 5:**
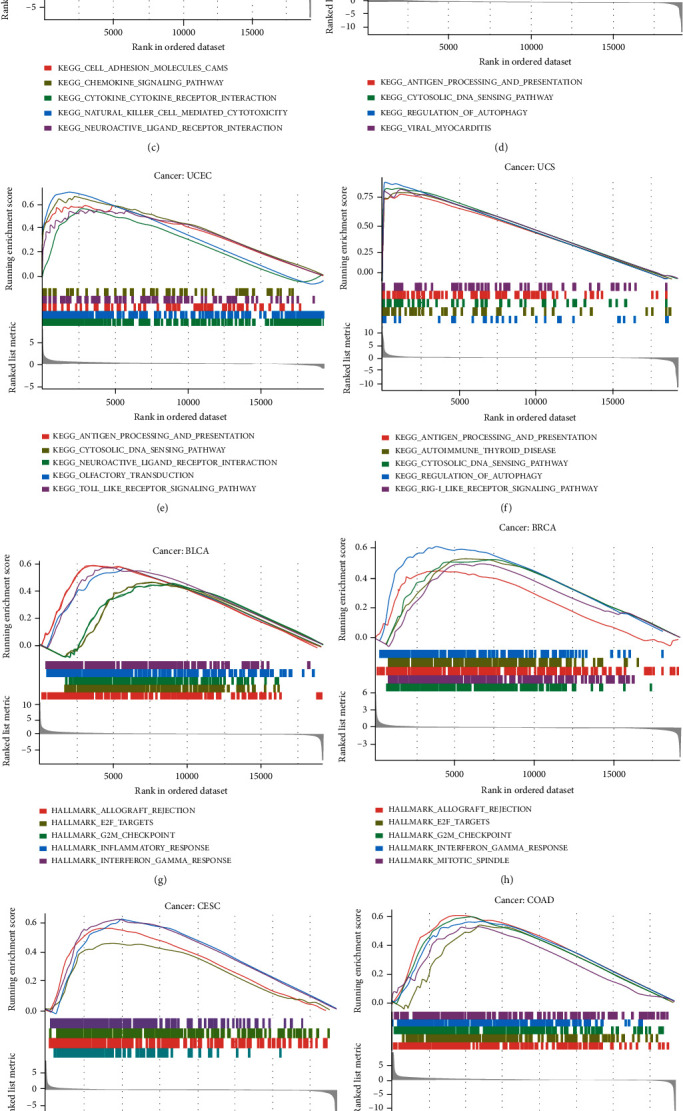
Gene set enrichment analysis of ADAR1 in the KEGG and HALLMARK gene sets. (a–f) KEGG and (g–l) HALLMARK.

To preliminarily explore the mechanism of ADAR1 in pan-cancer, we performed GSEA analysis using KEGG, HALLMARKER, and immunologic signature gene sets, respectively. The results indicated that ADAR1 was involved in a variety of signaling pathways. In KEGG, ADAR1 is closely related to pathways of cell adhesion, antigen processing, and chemokine expression (Figures 5(a)–5(f)). In HALLMARK sets, ADAR1was involved in the inflammatory and interferon pathways (Figures 5(g)–5(l)). These results suggested that ADAR1 may play an important role in regulating the tumor immune microenvironment.

### 3.5. Association of ADAR1 Expression with Immune Cell Infiltration, Immune Checkpoints, and Chemokines in Pan-Cancer

**Figure 6:**
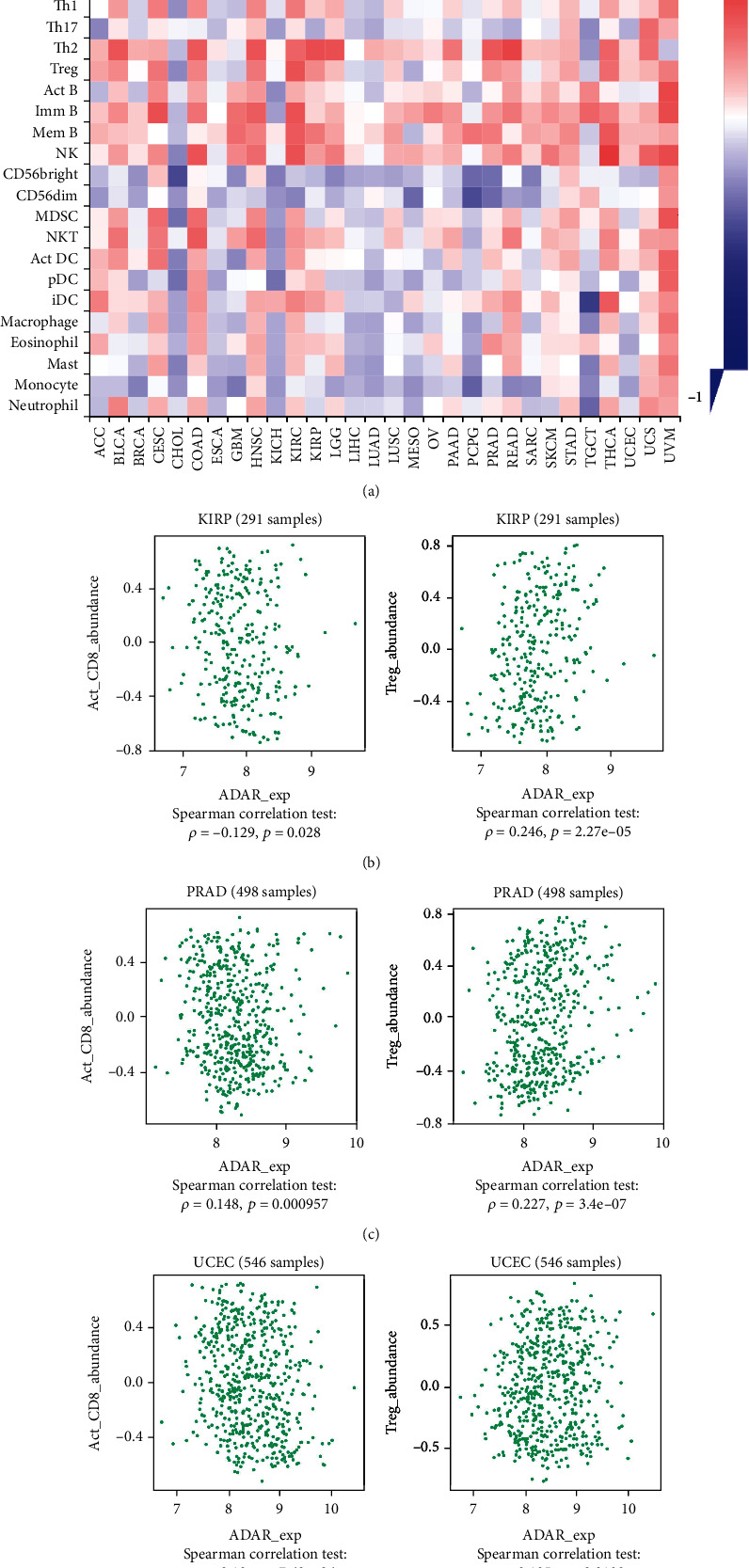
ADAR1 expression level and immune cell infiltration analysis. (a) Correlation between ADAR1 and the level of infiltration of indicator immune cells by TISDB database. (b) The association of ADAR1 with CD8^+^ T cell and Treg cell infiltration in KIRP. (c) The association of ADAR1 with CD8^+^ T cell and Treg cell infiltration in PRAD. (d) The association of ADAR1 with CD8^+^ T cell and Treg cell infiltration in UCEC.

**Figure 7:**
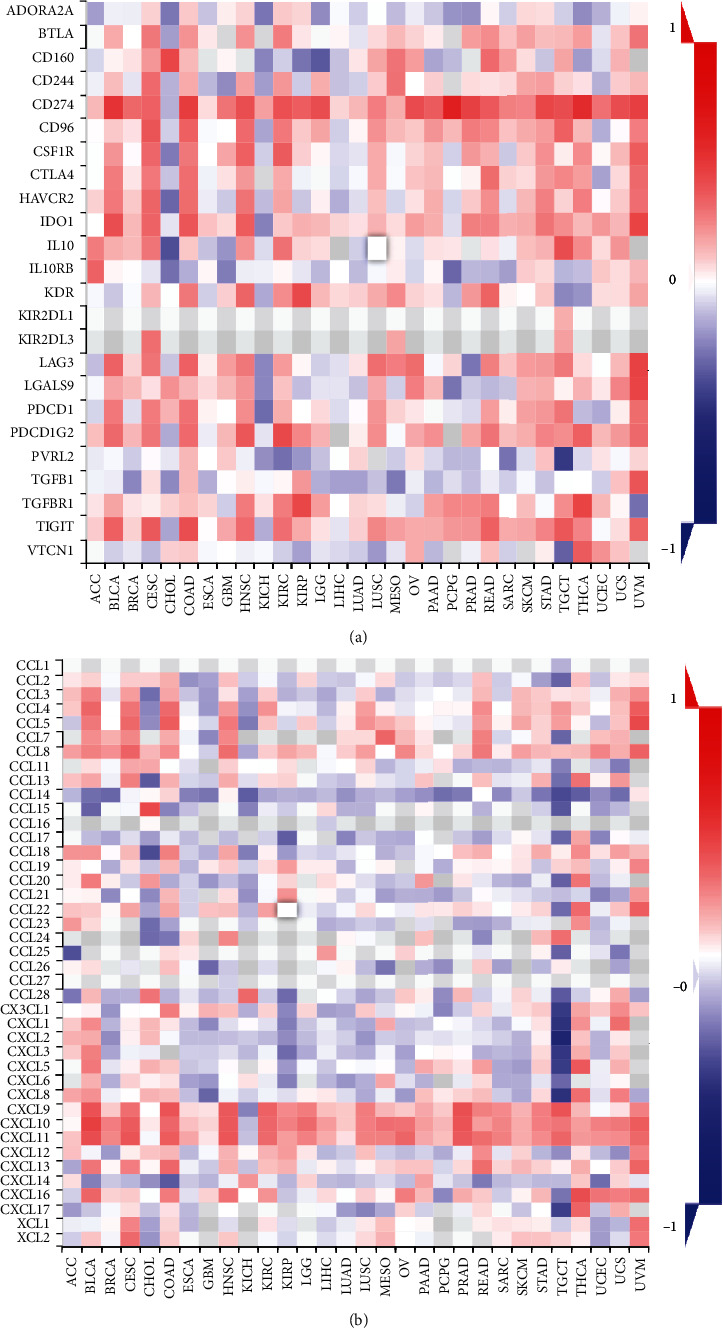
The relationship between ADAR1 and immunomodulatory molecules. (a) Association of ADAR1 with immune checkpoints in pan-cancer. (b) Association of ADAR1 with chemokines in pan-cancer.

Tumor-infiltrating immune cells are an important part of the complex microenvironment that regulates the development and progression of cancers [3]. Tumor-infiltrating immune cells are the main performers of tumor immune responses [19]. To explore whether ADAR1 expression level modulates the level of immune cell infiltration, we analyzed it by the TISIDB database (Figure 6(a)). The results showed that ADAR1 was negatively correlated with activated CD8^+^ T cells and positively correlated with Treg cells in KIRP, PRAD, and UCEC (Figures 6(b)–6(d)). Given that deficiencies in immune surveillance are an important cause of poor prognosis in various cancers. Tumors evade immune cell attack by exploiting multiple pathways, such as regulation of immune checkpoints and secretion of leukocyte chemokines. Therefore, we further investigated the relationship between ADAR1 and the above functions. The results showed that ADAR1 was positively correlated with the expression levels of immunosuppressive genes such as CD274, PDCD1, LAG3, CTLA4, TIGIT, CD96, and IDO1 in most tumors (Figure 7(a)). ADAR1 positively correlated with chemokines, such as CCL14 and CCL28 in most tumors (Figure 7(b)). These data suggested that ADAR1 may regulate the expression of immune checkpoints and chemokines to modulate the tumor immune microenvironment.

### 3.6. Correlation Analysis of ADAR1 and Tumor Stemness in Pan-Cancer

**Figure 8:**
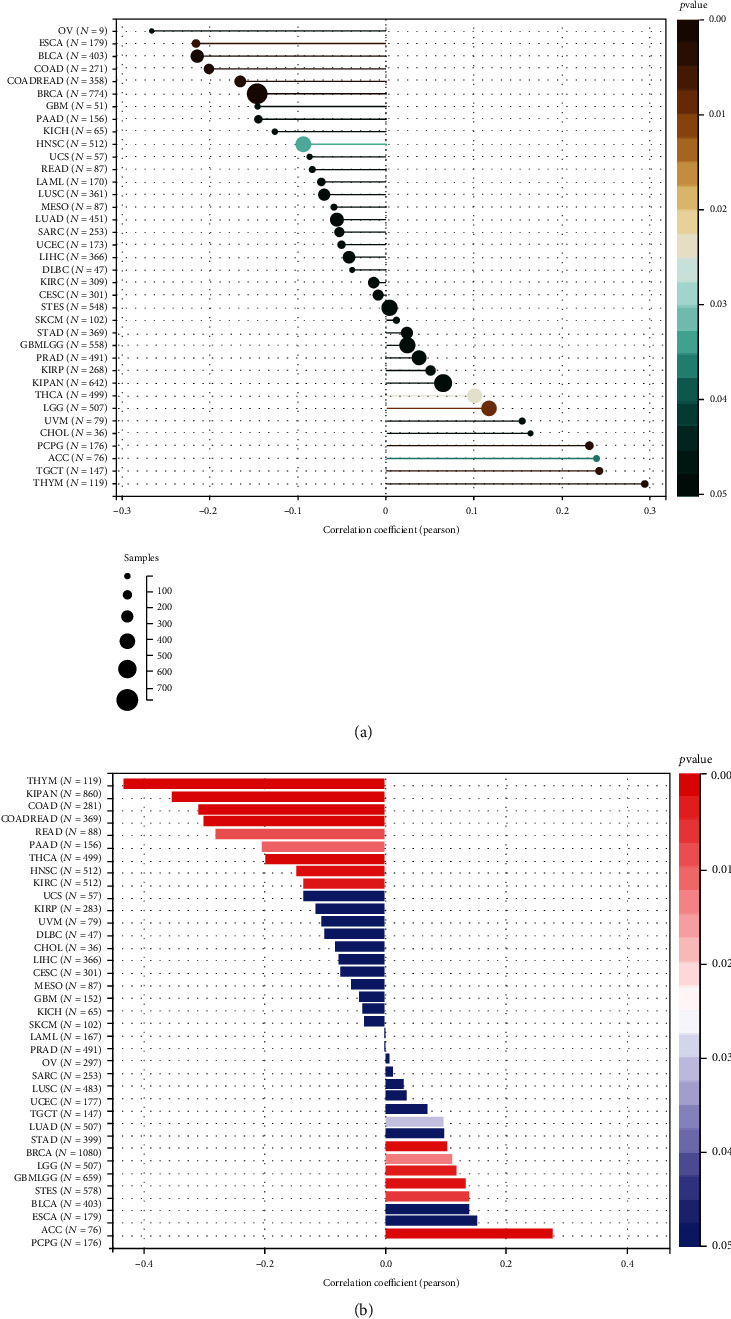
The correlation analysis of ADAR1 and tumor stem index in pan-cancer. (a) The relationship between ADAR1 and DNAss in pan-cancer. (b) The relationship between ADAR1 and RNAss in pan-cancer.

Stemness plays an important role in tumorigenesis and development [20]. We further analyzed the relationship between ADAR1 and tumor stemness. We further applied DNA stemness signature (DNAss) and RNA stemness signature (RNAss) to assess the role of ADAR1 on tumor stemness capacity. The results showed that ADAR1 expression levels were positively correlated with DNAss in STES, SKCM, STAD, GBM, PRAD, KIRP, KIPAN, THCA, LGG, UVM, CHOL, PCPG, ACC, TGCT, and THYM. In contrast, ADAR1 expression levels were negatively correlated with DNAss in CESC, KIRC, DLBC, LIHC, UCEC, SARC, LUAD, MESO, LUSC, LAML, READ, UCS, HNSC, KICH, PAAD, GBM, BRCA, COAD, BLCA, ESCA, and OV (Figure 8(a)). In addition, ADAR1 expression levels were positively correlated with RNAss in LAML, PRAD, OV, SARC, LUSC, UCEC, TGCT, LUAD, STAD, BRCA, LGG, STES, BLCA, ESCA, ACC, and PCPG. In contrast, ADAR1 expression levels were negatively correlated with RNAss in SKCM, KICH, GBM, MESO, CESC, LIHC, CHOL, DLBC, UVM, KIRP, UCS, KIRC, HNSC, THCA, PAAD, READ, COAD, KIPAN, and THYM (Figure 8(b)).

## 4. Discussions

Immune evasion plays a crucial role in tumor progression and metastasis, which is the theoretical basis of immunotherapy [21]. In this study, we revealed that ADAR1 was highly expressed in most cancers, such as BRCA, LUAD, ESCA, LUSC, STAD, CHOL, CESC, HNSC, UCEC, PCPG, BLCA, COAD, THCA, and LIHC. In addition, GSEA analysis revealed that ADAR1 may be involved in immune processes such as antigen presentation, cell adhesion, and chemokine expression. Moreover, we also found that ADAR1 was significantly associated with patient prognosis in pan-cancer. In addition, we further found through public data that ADAR1 may be involved in the regulation of immune cell infiltration, immune checkpoints, and chemokine expression in pan-cancer. Finally, we revealed that ADAR1 is involved in the regulation of tumor stemness in pan-cancer.

The biological behavior of cells is regulated by gene networks [22]. A previous report revealed that loss of ADAR1 rendered tumors highly sensitive to immunotherapy and overcame resistance to immune checkpoint inhibitors [9]. In this study, we performed GSEA analysis at the pan-cancer level, and the results showed that the high expression of ADAR1 was mainly related to immune-related effects. This further corroborates the complex role of ADAR1 in regulating tumor immunity and provides a theoretical basis for the development of immune drugs targeting ADAR1.

At present, there are a variety of approaches for tumor immunotherapy, including monoclonal antibody-based immune checkpoint inhibitors, cancer vaccines, and therapeutic antibodies. Here, we collected more than 40 common immune checkpoint genes, extracted these immune checkpoint genes separately, and calculated the correlation with our target gene expression. The results showed that the upregulation of ADAR1 is closely related to the immune checkpoints of various cancers and also has a significant correlation with the infiltration of immune cells. Combining ADAR1 with prognostic and significant associations in a variety of tumors, our study thus elucidated the potential role of ADAR1 in tumor immunology and its use as a cancer prognostic biomarker.

Recent studies suggest that ADAR1 may also be involved in promoting tumorigenesis. Downregulation of ADAR1 in breast cancer cells reduced cell proliferation, which was not seen in other cell lines [23]. It is unclear why some cancer cells depend on ADAR1 or why other cells are not sensitive to the loss of ADAR1. Elevated expression of IFN and ADAR1 has been observed in many cancer types [8]. Kung et al. found that ADAR1-dependent TNBC cell lines also exhibited elevated IFN-stimulated gene expression and reduction of IFNAR1 apparently rescued the proliferation defect of ADAR1 deficiency [24]. Ramirez-Moya et al. have confirmed that ADAR1-mediated A-to-I editing was one of the important pathways that promote thyroid cancer progression, and blocking RNA editing is a potential therapeutic target for thyroid cancer [25]. In this study, our pathway enrichment results showed that ADAR1 is associated with multiple validated pathways in multiple cancers, which further supports these previous findings. This suggested that ADAR1 plays a diverse role in tumors, and targeting ADAR1 is a promising treatment option for tumor. The data and relevant clinical information of this study are all from public databases and lack of in vivo and in vitro verification. We will further confirm the role of ADAR1 in tumors in subsequent studies.

In conclusion, we conducted a comprehensive evaluation of ADAR1 in pan-cancer, revealing that ADAR1 is a prognostic indicator for a variety of tumors and further exploring its implications in tumor immunity. ADAR1 may be a potential target for immunotherapy in a variety of tumors.

## Data Availability

The data in this study are available from the corresponding authors upon reasonable request.

## Supplementary Materials

Supplementary Materials Supplementary Figure 1: protein levels of ADAR1 in lung cancer. (A) Expression level of ADAR1 protein in lung cancer tissues. (B) Protein expression of ADAR1 in normal lung tissue.
