# Supplementary material for: A Comprehensive and Systematic Analysis Revealed the Role of ADAR1 in Pan-Cancer Prognosis and Immune Implications
Source: Dis Markers. 2023 Feb 21;2023:7620181. doi: 10.1155/2023/7620181 (PMC9974249; doi:10.1155/2023/7620181)
Supplement: Supplementary Materials — Supplementary Figure 1: protein levels of ADAR1 in lung cancer. (A) Expression level of ADAR1 protein in lung cancer tissues. (B) Protein expression of ADAR1 in normal lung tissue. [file 7620181.f1.pdf]

Supplementary Figure

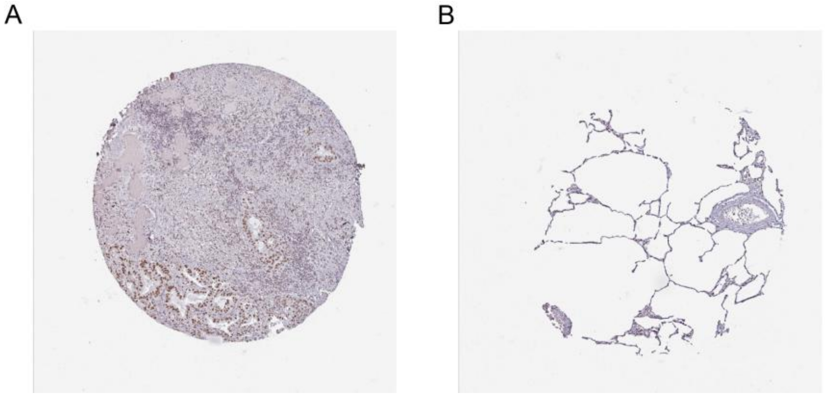

Supplementary Figure1. Protein levels of ADAR1 in lung-cancer

(A) Expression level of ADAR1 protein in lung cancer tissues. (B) Protein expression of ADAR1 in normal lung tissue.
